# Who Am I? Self-concept in Adults with Cystic Fibrosis: Association with Anxiety and Depression

**DOI:** 10.1007/s10880-024-10023-7

**Published:** 2024-07-09

**Authors:** Maggie Harrigan, Siobhain Mulrennan, Melanie Jessup, Phoebe Waters, Kellie Bennett

**Affiliations:** 1 Medical School, The University of Western Australia (UWA), Perth, WA Australia; 2 Institute for Respiratory Health (IRH), Perth, WA Australia; 3 Cystic Fibrosis Western Australia (CFWA), Perth, WA Australia; 4 Sir Chares Gairdner Hospital (SCGH) Adult Cystic Fibrosis Clinic, Perth, WA Australia; 5 School of Nursing, Midwifery and Social Work, The University of Queensland (UQ), Brisbane, QLD Australia

**Keywords:** Cystic fibrosis (CF), Self-concept, Identity, Mental health, CFTR modulator

## Abstract

Cystic Fibrosis (CF) is a progressive condition resulting in reduced lung function and strongly associated with elevated anxiety and depression symptoms. Self-concept refers to an individual’s overarching sense of identity, a positive level of which is widely associated with reduced anxiety and depression. There is a significant lack of self-concept research within CF. This study explores the association between self-concept and anxiety and depression in adults with CF. 64 adults living with CF in Western Australia completed validated online questionnaires (Generalised Anxiety Disorder-7, Patient Health Questionnaire-9, Tennessee Self-Concept Questionnaire 2: Short-Form) and consented to the collection of medical data. Descriptive, t-test, correlation and multiple regression analysis were undertaken. Higher levels of self-concept were associated with lower levels of anxiety and depression symptoms. Lower self-concept levels were a significant predictor of increased anxiety and depression symptoms after accounting for physical health status. Mean self-concept scores for those who required mental health intervention were significantly lower compared to those that did not. This study identifies a significant relationship between self-concept and anxiety and depression in adults with CF. Further research is required to establish causation and test the feasibility of self-concept interventions in reducing anxiety and depression symptoms.

## Introduction

Cystic Fibrosis (CF) is a progressive genetic disease resulting from cystic fibrosis transmembrane conductance regulator (CFTR) gene mutations and impacting more than 70,000 people globally (Cystic Fibrosis Foundation, 2023). It is a disease most renowned for reduced lung function but affects multiple organs, including the pancreas, gastrointestinal tract, liver and reproductive systems (Bell et al., 2015). Symptoms of anxiety and depression are between two to three times higher in individuals with CF than those without the disease (Quittner et al., 2014). This poses a significant challenge to enhancing health outcomes, given that suboptimal mental health within CF has been associated with worsening physical health outcomes and mortality (Fidika et al., 2014; Riekert et al., 2007; Schechter et al., 2021). With such significant consequences, there is a continuing need to understand and develop effective interventions to support the mental health of those living with CF.

Self-concept is rooted in psychoanalytical self-psychology theory: the belief that an individual’s sense of self determines how they function in all aspects of life (Kohut, 2009). Otherwise known as self-perception or self-identity, it refers to an individual’s internal sense of who they are as a person; how they would respond to asking themselves, *who am I*? This encompasses a comprehensive self-evaluation of characteristics, moral code, values, strengths, weaknesses, likes, dislikes, behaviours and roles over the multiple facets of physical, personal, emotional, social, academic and professional life (Baumeister, 1999; Shavelson et al., 1976). Wide agreement exists that self-concept is malleable and dynamic, manifesting from biological, environmental and social factors (Markus & Wurk, 1987). As an overarching concept, it incorporates various overlapping dimensions, such as self-esteem or self-worth (value you place on yourself), self-efficacy (belief in your abilities) and social self (sense of interpersonal closeness with others) (Ackerman, 2023; Kohut, 2009; Lee & Robbins, 1998).

Outside the CF context, positive self-concept is strongly linked with better physical, mental and social health, and in particular is associated with reduced symptoms of anxiety and depression within chronic illness (Butler et al., 2020; Fuentes et al., 2011; Gannotti et al., 2011; Hards et al., 2020; Kiropoulos et al., 2021; Morales-Sánchez et al., 2021; Norrington, 2021; Rodríguez et al., 2012). Despite the prevalence of anxiety and depression symptoms, and a range of unique pertinent disease factors, there is a significant lack of self-concept research within CF. For example, the rigorous CF daily treatment regime, stringent infection control guidelines precluding face-to-face contact between those with CF (Muther et al., 2018), and cystic fibrosis transmembrane conductance regulator (CFTR) modulator drugs, the most recent of which (elexacaftor/tezacaftor/ivacaftor, Trikafta®, Vertex Pharmaceuticals Incorporated) have demonstrated transformative quality-of-life and physical health outcomes (Finlay et al., 2022; Havermans & Duff, 2020; Schechter et al., 2021). A systematic review of self-concept research within CF reveals a limited body of data focussing primarily on self-efficacy regarding physical health management (Harrigan et al., 2024). Three studies specifically explore self-concept alongside anxiety and depression within a CF context, however, they focus on only one specific aspect of self-concept (emotional regulation self-efficacy, general self-efficacy, optimism), are predominantly conducted with an adolescent and young adult population and all highlight the need for further self-concept research within CF (Mitmansgruber et al., 2021; Oliver et al., 2019; Russell et al., 2021).

The primary aim of this research was to explore the relationship between self-concept and anxiety and depression symptoms within an adult CF context. The following hypotheses were tested within an adult CF sample:

1. Higher levels of self-concept are associated with lower levels of anxiety and depression symptoms.
2. Lower self-concept levels are a significant predictor of increased anxiety and depression symptoms after accounting for physical health status.
3. Mean self-concept scores for those requiring mental health intervention are significantly lower compared with those that do not.

## Materials and Methods

### Procedure

A cross-sectional quantitative design was employed for this study, using self-report questionnaires and medical records data. The study took place during 2021 with 183 non-transplanted adults with CF living in Western Australia. This study received approval from The University of Western Australia (UWA) Human Research Ethics Committee (Ref: 2021/ET000534). Participants were recruited from Sir Charles Gairdner Hospital Adult CF Clinic and the Institute for Respiratory Health. The CF Clinic serves as the primary point of multi-disciplinary respiratory care for all adults with CF living in Western Australia up until the point of lung transplantation, at which point of care is transferred to another hospital. The CF clinic works closely with the Institute for Respiratory Health (IRH), a non-government respiratory research organisation. Initial screening of potential participants was conducted by a respiratory consultant, who is a member of the research team and works across both organisations. Initial screening applied the following inclusion criteria:

- Aged 18 + years old (adult-based project and organisations).
- Diagnosis of CF.
- Current patient of Sir Charles Gairdner Hospital Adult CF Clinic (excludes post lung-transplant CF patients).
- No known intellectual disability, psychosis, or significant mental illness that would deem participation inappropriate or distressing.
- Able to read English.

The inclusion criteria were met by 180 people, of which 179 had contact details available, and were initially contacted by a member of CF clinic or IRH recruitment officer independent from the research team. Brief details of the study were provided, and consent sought for further contact from the research team. Participants who agreed to participate were given access to the online survey via the UWA Data Qualtrics Platform. The survey included an online consent form that participants had to complete before completing the survey. To ensure socioeconomic inclusivity, participants were given the option to complete the survey via hard copy or telephone. However, all participants opted for online completion. Participants were advised to complete the survey at least two weeks post-acute illness due to the potential for this to impact answers and not represent usual mood. All participants were provided with written information on support options should they experience any emotional discomfort during or after the survey. Where indicated, appropriate follow-up was arranged in consultation with participants and in coordination with the CF Clinic, as was fully explained during the consent process.

### Measures

#### Demographics

Basic demographic information was collected to provide an insight into socioeconomic characteristics, namely, age, gender, relationship status, living situation, home ownership status, income, qualifications and employment status.

#### Physical health

Key CF physical health markers were obtained from the previous 24 months of medical records to provide insight into the physical health of participants and to explore any correlations and account for confounders between physical health, anxiety and depression.

The survey included the following self-report measures:

*Tennessee Self-Concept Questionnaire: 2 Short-Form (TSCS:2 short-form, adult) *(Fitts & Warren, 1996)

TSCS: 2 short-form consists of twenty statements relating to self-concept and scored on a five-point Likert scale. The resulting score represents overarching self-concept, with higher scores representing more positive self-concept. Raw scores are converted into percentile-based normative data T-scores to aid categorisation of self-concept into very low (30 or less), low (31–40), average (41–59), high (60–69) and very high (70 +). Caution is recommended regarding very high scores which can in fact indicate an unrealistic positive self-view and psychological distress. This questionnaire was developed and validated within an adult chronic illness setting and has been used in previous studies to measure self-concept for adults with Multiple Sclerosis and Cerebral Palsy (Gannotti et al., 2011; Kiropoulos et al., 2021). A licensing agreement was obtained from Western Psychological Services (WPS) in order to use this questionnaire.

*Generalised Anxiety Disorder (GAD-7) *(Spitzer et al., 2006)

GAD-7 consists of seven items relating to symptoms of anxiety across the past two weeks and is scored on a four-point Likert scale of symptom frequency. Total scores are categorised as normal range (0–4), mild (5–9), moderate (10–14) and severe (15 +). This is a publicly available, highly validated questionnaire and is recommended specifically for people with CF to assess the presence of anxiety symptoms (Quittner et al., 2016). International CF guidelines recommend supportive intervention and psychoeducation for mild scores, and clinical assessment and intervention for moderate to severe scores (Quittner et al., 2016).

*Patient Health *Questionnaire* 9 (PHQ-9*) (Spitzer et al., 1999)

PHQ-9 consists of nine items relating to symptoms of depression across the past two weeks. It is scored on a four-point Likert scale of symptom frequency. Total scores are categorised as normal range (0–4), mild (5–9), moderate (10–14), moderately severe (15–19) and severe (20 +). This is a publicly available, highly validated questionnaire, recommended specifically for people with CF to assess the presence of depression symptoms (Quittner et al., 2016). International CF guidelines recommend supportive intervention and psychoeducation for mild scores, and clinical assessment and intervention for moderate to severe scores (Quittner et al., 2016).

### Data analysis

Data analysis was carried out using IBM SPSS version 23.0 software package (IBM Corp, 2015). Scores from the GAD-7 were used to measure anxiety symptoms, and PHQ-9 scores were used to measure depression symptoms as dependent variables. TSCS-2 (short-form) scores were used to measure overarching self-concept as an independent variable. The various physical health data collected were also analysed as independent variables. Initial descriptive data analysis was conducted to illustrate socioeconomic demographics, physical health characteristics, and levels of self-concept, anxiety and depression. Correlation and hierarchical multiple regression analysis was conducted to identify which independent variables had statistically significant correlations with anxiety and/or depression, and to determine the contribution of self-concept to the prediction of anxiety and depression after accounting for physical health status. Correlation direction and strength was determined based upon interpretation of the r_s_ value (Evans, 1996).

## Results

### Sample characteristics

Sixty-four people participated in this study and answered every survey item. Table 1 provides an overview of sample demographics, demonstrating diversity of gender, age, social, education and economic characteristics. Table 2 provides an overview of physical health data obtained. In line with contemporary CF research, a comprehensive range of physical health measures were collected to provide a more accurate representation of physical health status (Schechter et al., 2021).

**Table 1:** Participant characteristics (N = 64): Demographics

Social	n (%)	Education and economic	n (%)
*Gender*		*Home ownership*	
Female	28 (43.75)	Owned outright	12 (18.75)
Male	35 (54.69)	Owned with mortgage	34 (53.13)
Non-binary	1 (1.56)	Rented	13 (20.31)
		Other	5 (7.81)
*Age*		*Employment status*	
18–25	14 (21.88)	Unemployed	10 (15.63)
26–33	15 (23.44)	Full-time	34 (53.13)
34–41	13 (20.31)	Part-time	13 (20.31)
42–57	18 (28.13)	Temporary/casual	7 (10.94)
58+	4 (6.25)		
*Location*		*Highest schooling*	
Metro	50 (78.13)	Year 10	11 (17.19)
Regional	14 (21.88)	Year 11	8 (12.50)
		Year 12	45 (70.31)
*Marital status*		*Qualifications*	
Divorced	2 (3.13)	Nil	10 (15.63)
Married	33 (51.56)	Studying for first qualification	3 (4.69)
Never Married	27 (42.19)	Trade cert/apprenticeship	16 (25.00)
Separated	1 (1.56)	Other (e.g. diploma/degree)	35 (54.69)
Widowed	1 (1.56)		
*Relationship status*		*Weekly gross personal income*	
In a relationship	51 (79.69)	$0–$499	14 (21.88)
Single	13 (20.31)	$500–$799	10 (15.63)
		$800–$1249	10 (15.63)
		$1250–$1749	9 (14.06)
		$1750–$2999	14 (21.88)
*Living situation*		$3000+	6 (9.38)
Alone	11 (17.19)		
Not alone	53 (82.81)		

% rounded to two decimal places

**Table 2:** Participant characteristics (N = 64): Physical health

Physical health variables	*n* (%)	Physical health variables	*n* (%)
*Lung function (FEV1%) *(mean 68.4, SD = 21.1)		*No. of oral antibiotic courses (POAB) (24 months)* (mean 2.9, = SD 2.3)	
< 40	5 (7.81)	Nil	10 (15.63)
40–59	18 (28.13)	1–3	33 (51.56)
60–80	15 (23.44)	4–6	17 (26.56)
> 80%	23 (35.94)	7+	4 (6.25)
Unknown	3 (4.69)		
*Body Mass Index* (mean 25.8, SD = 6.4)		*No. of co-morbidities* (mean 6.0, SD = 2.3)	
< 18.5	4 (6.25)	1–3	9 (14.06)
18.5–24.9	29 (45.31)	4–6	32 (50.00)
25–29.9	18 (28.13)	7–9	19 (29.69)
30+	13 (20.31)	10+	4 (6.25)
*Total pulmonary exacerbations (PEx) (24 months)* (mean 4.2, SD = 3.4)		*No. of regular medications* (mean 8.3, SD = 4.4)	
Nil	8 (12.50)	1–5	17 (26.56)
1–3	23 (35.94)	6–10	29 (45.31)
4–6	19 (29.69)	11–15	14 (21.88)
7–9	8 (12.50)	16+	4 (6.25)
10+	6 (9.38)		
*No. of intravenous antibiotic courses (IVABs) (24 months)* (mean 1.3, SD = 1.7)		*No. of cultured pathogens* (mean 2.3, SD = 1.9)	
Nil	33 (51.56)	None	11 (17.19)
1–3	24 (37.50)	1–3	39 (60.94)
4–6	7 (10.94)	4–6	12 (18.75)
		7–9	2 (3.13)
*Lung transplant status*		*Pathogen growth*	
Nil	55 (85.94)	Non-cepacia	59 (92.19)
Input (referred, under review, waitlisted)	9 (14.06)	B. cepacia or M. abscessus	5 (7.81)
*Hospitalisations*		*CFTR modulator status*	
Nil	38 (59.38)	Nil	19 (29.69)
1	12 (18.75)	Ivacafto	3 (4.69)
2	10 (15.63)	Kalydeco	3 (4.69)
3+	4 (6.25)	Orkambi	8 (12.50)
		Symdeko	20 (31.25)
		Trikafta (compassionate access)	11 (17.19)

*SD* standard deviation, *Mean and SD* 1 decimal place, % *rounded to nearest two decimal places, FEV1%* forced expiratory volume in 1 second percent predicted, *Non-cepecia* includes all strains of Pseudomonas, *B.cepacia* Burkholderia cepacian, *M. abscessus* Mycobacterium abscessus, *CFTR* cystic fibrosis transmembrane conductance regulator

### Levels of self-concept, anxiety and depression

**Fig. 1:**
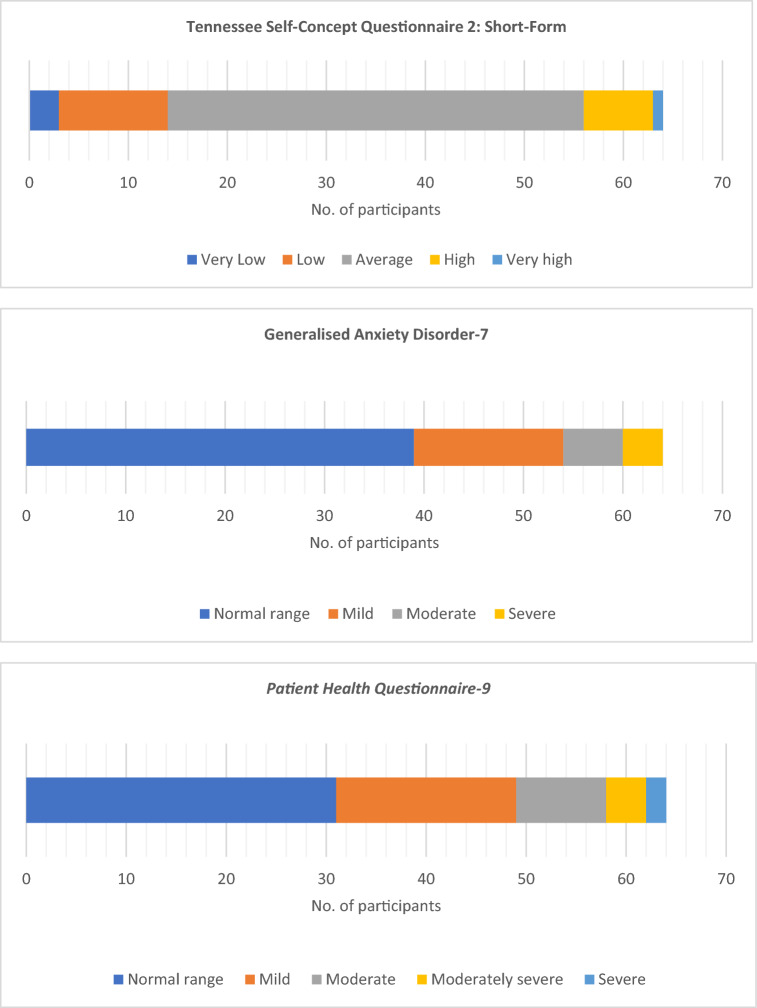
Self-concept, anxiety and depression scores by category (N = 64)

Figure 1 depicts self-concept, anxiety and depression scores by category. Self-concept scores ranged from 27 to 71 (possible range 19–79) with a mean score of 48.31 (*SD* = 10.40). Low or very low self-concept scores were recorded by 21.88% of participants. Anxiety scores ranged from 0 to 19 (possible range 0–21) with a mean score of 4.50 (*SD* = 4.80). Mild to severe scores for anxiety were found in 39.07% of participants. Depression scores ranged from 0 to 24 (possible range 0–27) with a mean score of 6.00 (*SD* = 5.47). Mild to severe scores for depression were identified in 51.57% of participants.

### Association between self-concept and anxiety and depression

Given lack of normal distribution demonstrated through Shapiro–Wilk testing and examination of histograms, Spearman’s rho correlation coefficient was applied to calculate the strength and direction of linear relationships between self-concept and anxiety and depression. Self-concept had a moderate negative correlation with anxiety (*r*_*s*_*—*0.581), and a strong negative correlation with depression (*r*_*s*_*—*0.649). Self-concept had a statically significant linear correlation with anxiety and depression (*p* < .001, 2-*tailed)*.

### Self-concept as a predictor of anxiety and depression

Correlation analysis was first conducted to explore linear correlations between physical health markers and anxiety and depression, to indicate significant correlations between physical health variables and self-concept. Given lack of normal distribution, Spearman’s rho correlation coefficient was again applied. Both frequency of pulmonary exacerbations and oral antibiotic courses demonstrated statistical significant (*p* < .001, 2-*tailed)* (Table 3).

**Table 3:** Correlations between physical health variables and anxiety and depression (N = 64)

Independent variables	Anxiety symptoms (GAD-7)		Depression symptoms (PHQ-9)	
	Spearman’s rho	*p* value	Spearman’s rho	*p* value
FEV1%	0.008		− 0.054	
Body mass index	− 0.099		− 0.023	
Total pulmonary exacerbations (PEx) (24 months)	0.283	**	0.407	**
Intravenous antibiotic courses (24 months)	0.086		0.204	
No. of oral antibiotic courses (24 months)	0.335	**	0.435	***
Hospitalisations (24 months)	0.150		0.181	
Co-morbidities	0.101		0.126	
No. of regular medications	0.042		0.046	
No. of cultured pathogens	0.039		0.075	

*p < .05; **p < .01; ***p < .001; (2-tailed)

Hierarchal multiple regression analysis (MRA) was conducted to determine if self-concept accounted for a significant proportion of the variance in anxiety and depression, beyond that accounted for by physical health status, as measured by pulmonary exacerbations. The inclusion of frequency of pulmonary exacerbations was based on established research findings, which consistently identify a relationship with anxiety and depression (Graziano et al., 2020; Quittner et al., 2014; Schechter et al., 2021). Oral antibiotic use was not included in the model as it has a high inter-correlation with pulmonary exacerbations and as it is often used as a surrogate marker of pulmonary exacerbations.

Before interpreting the output of the MRA, a number of assumptions were tested. Histogram, stem-and-leaf and Shapiro–Wilk testing indicated that self-concept was normally distributed and free from univariate outliers. Frequency of exacerbations, depression and anxiety were positively skewed and underwent arithmetic transformation (square root and log 10) resulting in approximate normality of distribution (demonstrated through histogram inspection and Shapiro–Wilk or Kolmogorov–Smirnov normality testing). Inspection of the normal probability plot of standardised residuals and scatterplot of standardised residuals against standardised predicted values indicated the assumptions of normality, linearity and homoscedasticity of residuals were met. Normality plots with Shapiro–Wilk testing revealed normal distribution amongst residuals, and that their relationship with the predicted values was linear. Mahalanobis distance did not exceed the critical *χ*^2^ for *df* = 2 (*at α* = .001) of 13.82 for any cases in the data file, indicating that multivariate outliers were not of concern. Relatively high tolerances for both predictors in the regression model (self-concept and frequency of pulmonary exacerbations) indicated that multicollinearity would not interfere with MRA reliability.

On step 1 of the hierarchical MRA for predicting anxiety, frequency of pulmonary exacerbations accounted for a non-significant 5.40% of the variance (*R*^2^ = .054,* F*(1, 51) = 2.897*, p* = .095). On step 2, self-concept was added to the regression equation, and accounted for an additional 27.5% of the variance (*R*^*2*^* change* = 0.275*, F change* (1, 50) = 20.513, *p* < .001)*.* In combination, the two predictors accounted for a significant 32.9% of variance in the prediction of anxiety symptoms *(R*^2^ = .329*, adjusted R*^2^ = .302*, F*(2, 50) = 12.260*, p* < .001*),* which by Cohen’s (1998) convention is considered a large effect (*f*^2^ = .450). Self-concept contributed statistically significant unique variance (sr^2^ = .276) to the prediction of anxiety even after accounting for the contribution of frequency of pulmonary exacerbations.

On step 1 of the hierarchical MRA for predicting depression, frequency of pulmonary exacerbations accounted for a significant 17.2% of the variance (*R*^2^ = .172*, F*(1, 62) = 12.886*, p* = .001). On step 2, self-concept was added to the regression equation, and accounted for an additional 32.9% of the variance (*R*^*2*^* change* = *0.329, F change* (1, 61) = 40.213,* p* < .001*).* In combination, the two predictors accounted for a significant 50.10% of the variance in depression (*R*^2^ = .501, adjusted* R*^2^ = .485,* F*(2, 61) = 30.625*, p* < .001)*,* which by Cohen’s (1998) convention is considered a large effect (*f*^2^ = 1.004*).* Self-concept contributed statistically significant unique variance (*sr*^2^ = .329) to the prediction of depression even after accounting for the significant contribution of frequency of pulmonary exacerbations.

Significant levels (Sig.), unstandardised (*B)* and standardised (*β*) regression coefficients, and squared semi-partial (or ‘part’) correlations (sr^2^*)* for predictors on each step of the hierarchal MRA for predicting anxiety and depression are reported in Table 4.

**Table 4:** Regression modelling for predicting anxiety and depression symptoms (*N* = 64)

Variable	*B* (95% CI)	*β*	Sig	sr^*2*^
Predicting anxiety symptoms				
*Step 1*				
Total pulmonary exacerbations (PEx) (24 months)	.096 (− .017, .210)	.232	.095	.054
*Step 2*				
Total pulmonary exacerbations (PEx) (24 months)	.045 (− .054, .144)	.108	.367	.011
Self-concept	− .020 (− .029, − .011)	− .539	< .001***	.276

Predicting depression symptoms				
*Step 1*				
Total pulmonary exacerbations (PEx) (24 months)	.517 (.229, .805)	.415	0.001***	.172
*Step 2*				
Total pulmonary exacerbations (PEx) (24 months)	.315 (.081, .550)	.253	0.009***	.060
Self-concept	− .069 (− .091, − .047)	− .596	0.000***	.329

Rounding to 3 decimal places. CI = confidence interval, *t* = *t*-statistic, Sig. = significant levels (*p < .05; **p < .01; ***p < .001), B = unstandardised regression coefficient, *β* = standardised regression coefficient, *sr*^2^ = squared semi-partial correlation

### Mean self-concept scores in sub-set of participants requiring mental health intervention compared to participants who did not require mental health intervention

International CF guidelines recommend supportive intervention, psychoeducation or clinical assessment and intervention for people scoring 5+ on either GAD-7 (anxiety symptoms) or PHQ-9 (depression symptoms) (Quittner et al., 2016). The mean self-concept scores of participants with anxiety and/or depression scores indicating the need for some form of mental health intervention (n = 36) were compared to scores from participants who did not require any form of mental health intervention (n = 28). After confirming normal distribution via Shapiro–Wilk testing and equality of variance via Levene’s test, independent sample *t*-testing (with a 95% confidence interval) confirmed that the mean self-concept score for the group requiring intervention was significantly lower (*M* = 43.69, *SD* = 9.46) (*t*(62) = 4.64*, p* < .001) than the group not requiring intervention (*M* = 54.25, *SD* = 8.45), with a mean difference of 10.56 (95% CI 6.01 to 15.11)*.*

## Discussion

This cross-sectional quantitative study explored the relationship between self-concept and anxiety and depression symptoms within an adult CF context. Aligning with research outside of CF (Butler et al., 2020; Fuentes et al., 2011; Gannotti et al., 2011; Hards et al., 2020; Kiropoulos et al., 2021; Morales-Sánchez et al., 2021; Norrington, 2021; Rodríguez et al., 2012), and as hypothesised, higher levels of self-concept were associated with lower levels of anxiety and depression symptoms, lower self-concept levels were a significant predictor of increased anxiety and depression symptoms, after accounting for physical health status, and mean self-concept scores for those requiring mental health intervention were significantly lower compared with those that did not.

The heterogeneity of the CF community is well represented by the physical and socioeconomic diversity of the sample (Tables 1, 2). Consistent with other self-concept studies within an adult physical chronic disease setting (e.g. Multiple Sclerosis and Cerebral Palsy) (Gannotti et al., 2011; Kiropoulus et al., 2021), the majority of participants (77%) had healthy self-concept scores (Fig. 1). While this is reassuring, a notable proportion (almost one quarter) had low or very low self-concept scores indicative of a negative sense of self (Fitts & Warren, 1996).

The prevalence of mild to severe symptoms of depression and anxiety identified by the current study (Fig. 1) is much higher than that identified in 2014 during ‘The International Depression Epidemiological Study’ (TIDES) (Quittner et al., 2014), and when the same recruitment pool of participants were surveyed with GAD-7 and PHQ-9 in 2018 during regular CF clinic visits (*n* = 125) (Harrigan et al., 2019). The COVID-19 pandemic provides one potential explanation for this increased prevalence, with a significant increase in the prevalence of anxiety and depression globally (World Health Organisation, 2023). Sample bias may also be a factor, with those experiencing anxiety and depression symptoms more likely to have interest and participate in a mental health study that is not part of routine CF clinical care. Caution should therefore be applied to the prevalence of anxiety and depression symptoms reported.

A significant relationship between self-concept and anxiety and depression is highlighted. Results indicate that adults with higher levels of self-concept had lower symptoms of anxiety and depression. Conversely, adults with low levels of self-concept had higher levels of anxiety and depression. Additionally, self-concept played a vital role in contributing to levels of depression and anxiety in adults with CF, even after controlling for the impact of physical health status. This study provides insight into the relationship between self-concept, physical health, depression and anxiety in adult CF participants. A controlled study is recommended in order to establish causality between the variables and to test the feasibility of targeted self-concept interventions at reducing levels of anxiety and depression for adults living with CF. There are a range of existing generic evidence-based strategies to improve self-concept which could be drawn upon, such as psychoeducation, building positive relationships, goal setting, self-care and strengths-based approaches that recognise and utilise personal and relational strengths (Headspace, 2023; National Health Service, 2023).

While the prevalence of anxiety and depression symptoms for those living with CF is now well recognised (Quittner et al., 2014), and the Cystic Fibrosis Foundation (CFF) and European Cystic Fibrosis Society (ECFS) mental health guidelines have significantly increased mental health awareness and care (Quittner et al., 2016), there are a limited number of efficacy-tested psychotherapeutic interventions to specifically prevent and treat anxiety and depression in adults with CF (Friedman et al., 2022; Verkleij et al., 2012; Bathgate et al., 2022; O’Hayer et al., 2021). There are currently four efficacy-tested CF-specific interventions to reduce anxiety and depression that have demonstrated feasibility, acceptability and preliminary efficacy, with large-scale randomised control trials underway (CF-CBT, E-Health CF-CBT, CALM, ACT with CF) (Friedman et al., 2022; Verkleij et al., 2012; Bathgate et al., 2022; O’Hayer et al., 2021). Interestingly, these interventions all contain elements of self-concept exploration and promotion (Friedman et al., 2022; Verkleij et al., 2012; Bathgate et al., 2022; O’Hayer et al., 2021). In particular, self-concept is an intrinsic feature of acceptance and commitment therapy (ACT), utilised by the ACT with CF intervention (O’Hayer et al., 2021; Hayes et al., 2012). ACT with CF explores personal values and self-identity as a means of promoting psychologically flexibility and therapeutic change, and has demonstrated promising pilot data (Bathgate et al., 2022). Arguably, self-concept is particularly worthy of further research during the current era of rapidly evolving CFTR modulator care, with many of those living with CF having to re-calibrate many aspects of self, surrounding identity, life goals and life expectancy (Middleton et al., 2023; Heijerman et al., 2019; Balfour-Lynn & King, 2022). As CF treatment options continue to evolve, clinicians must ensure psychological interventions keep pace with the needs of the CF community if new treatments are to be fully optimised (Middleton et al., 2023; Heijerman et al., 2019; Balfour-Lynn & King, 2022).

There are several limitations of this research that must be acknowledged. The cross-sectional design of this study limits the ability to specify the direction of relationships between dependent and independent variables, and the small sample size limits generalizability and may contain bias towards those experiencing anxiety and depression. Furthermore, while participants were advised to wait at least two weeks post-acute illness before completing the survey, given the potential temporary impact on anxiety and depression symptoms, medical records were not used to verify participant compliance with this advice.

## Conclusions

This study highlights a significant negative relationship between self-concept and anxiety and depression after controlling for physical health status. Further research to establish causality between variables and to test the feasibility of targeted self-concept interventions to reduce levels of anxiety and depression for adults living with CF is recommended.

## Data Availability

A data set of full statistical analysis and outputs relating to this paper is securely stored in the UWA Institutional Research Data Store and can be made available upon request to the corresponding author. Not all original data will be shared in order to protect the privacy of research participants.
